# A Model of Oxidative Stress Management: Moderation of Carbohydrate Metabolizing Enzymes in SOD1-Null *Drosophila melanogaster*

**DOI:** 10.1371/journal.pone.0024518

**Published:** 2011-09-01

**Authors:** Kristine E. Bernard, Tony L. Parkes, Thomas J. S. Merritt

**Affiliations:** 1 Department of Biology, Laurentian University, Sudbury, Ontario, Canada; 2 Department of Biology and Chemistry, Nipissing University, North Bay, Ontario, Canada; 3 Department of Chemistry and Biochemistry, Laurentian University, Sudbury, Ontario, Canada; Universidade Federal do Rio de Janeiro, Brazil

## Abstract

The response to oxidative stress involves numerous genes and mutations in these genes often manifest in pleiotropic ways that presumably reflect perturbations in ROS-mediated physiology. The *Drosophila melanogaster* SOD1-null allele (*cSODn108*) is proposed to result in oxidative stress by preventing superoxide breakdown. In SOD1-null flies, oxidative stress management is thought to be reliant on the glutathione-dependent antioxidants that utilize NADPH to cycle between reduced and oxidized form. Previous studies suggest that SOD1-null *Drosophila* rely on lipid catabolism for energy rather than carbohydrate metabolism. We tested these connections by comparing the activity of carbohydrate metabolizing enzymes, lipid and triglyceride concentration, and steady state NADPH:NADP^+^ in SOD1-null and control transgenic rescue flies. We find a negative shift in the activity of carbohydrate metabolizing enzymes in SOD1-nulls and the NADP^+^-reducing enzymes were found to have significantly lower activity than the other enzymes assayed. Little evidence for the catabolism of lipids as preferential energy source was found, as the concentration of lipids and triglycerides were not significantly lower in SOD1-nulls compared with controls. Using a starvation assay to impact lipids and triglycerides, we found that lipids were indeed depleted in both genotypes when under starvation stress, suggesting that oxidative damage was not preventing the catabolism of lipids in SOD1-null flies. Remarkably, SOD1-nulls were also found to be relatively resistant to starvation. Age profiles of enzyme activity, triglyceride and lipid concentration indicates that the trends observed are consistent over the average lifespan of the SOD1-nulls. Based on our results, we propose a model of physiological response in which organisms under oxidative stress limit the production of ROS through the down-regulation of carbohydrate metabolism in order to moderate the products exiting the electron transport chain.

## Introduction

A single gene often affects many phenotypes through direct or indirect pleiotropic effects mediated through the gene products and/or substrates. While we often assume that a mutation will primarily cause disruptions in the immediate pathway of the gene product, alteration of a primary pathway may also induce changes in interacting pathways. Cumulatively the relationships and interactions among pathways form a network and determine the overall condition of the organism. Elucidating these network interactions is important in the verification of the underlying etiology of mutant phenotypes, and shapes our fundamental understanding of not only biological networks and pathways but also their evolution.

Mutation of genes involved in the oxidative stress response often manifest such pleiotropic impacts. Reactive oxygen species (ROS) collectively refers to the damaging by-products of aerobic metabolism, e.g. superoxide, hydrogen peroxide, and the hydroxyl radical. The lack of clearance of ROS is referred to as oxidative stress, and can result in oxidative damage, leading to physiological dysfunction and many types of age-related disease [1], [2], [3], [4], [5], [6], [7], [8], [9], [10]. The enzymes superoxide dismutase (SOD), catalase (CAT), and glutathione-dependant antioxidants have an evolutionarily conserved role in the metabolism and removal of ROS [reviewed in [11]. Mutants deficient in ROS metabolism display reduced longevity, acute sensitivity to stress, including but not limited to, oxidative stress, and a variety of whole-organism phenotypes. These phenotypes are hypothesized to result from deficiencies in ROS-metabolism and therefore perturbations in ROS-mediated physiological processes.

The ROS-scavenging enzyme SOD acts as the first line of defense against superoxide released from the electron transport chain (ETC). Superoxide released into the cytosol and mitochondrial intermembrane space is scavenged by SOD1 (CuZn or cytosolic SOD), and that released into the mitochondrial matrix is scavenged by SOD2 (Mn or mitochondrial SOD)[reviewed in [11]. *Drosophila melanogaster* deficient for SOD1 show a complex phenotypic syndrome characterized by an 80–90% reduction in longevity, complete male sterility, bloated abdomens, crinkled wings, an increased sensitivity to transition metals and RO-generating agents such as paraquat, [12], [13], and anomalies in locomotory and other behavioural functions [14]. SOD2-null mutants are characterized by a severely reduced adult lifespan [15], [16], [17], sensitivity to paraquat [16], decreased olfactory function [15], decreased function of mitochondrial enzymes [15], [16], and decreased cardiac performance and locomotor ability [18]. Interestingly, it has been found that switching SOD2-nulls from normoxia to hypoxia rescues early mortality; this, however, is not the case for SOD1-nulls [19].

SOD1-null mutants also have an increased dependency on the redox buffering capacity provided by the antioxidant tripeptide, glutathione (GSH) [20]. In dipteran insects, cycling between the reduced (GSH) and oxidized (GSSG) forms of glutathione is dependent on the thioredoxin (Trx) system, which includes a thioredoxin reductase and peroxidase [13], [21], [22], [23]. Much like glutathione reductase in other animals, the Drosophila thioredoxin reductase is able to convert oxidized glutathione (GSSG) to the reduced form (GSH), which then acts as an efficient reducing agent and ROS scavenger. The reducing cofactor NADPH is essential to the function of the thioredoxin system due to its donation of the hydrogen required for the reduction of oxidized glutathione [21]. It is therefore reasonable to speculate that changes which increase NADPH production would be beneficial to SOD1-null organisms. In fact, an increasing volume of evidence suggests that a variety of organisms cope with conditions of oxidative stress via increased reliance upon ancillary enzyme systems [15], [16] such as those that increase the production of NADPH [24], [25], [26], [27], [28], [29], [30], [31].

In addition to this role in managing oxidative stress, NADPH is also required in the synthesis of lipids [32]. This utilization of NADPH has, however, only been indirectly observed, as most studies have measured only the activity of NADP^+^-reducing enzymes and not actual NADPH:NADP^+^ concentrations [32], [33], [34]. Four key enzymes are responsible for the production of NADPH in the cytosol: malic enzyme (MEN) and isocitrate dehydrogenase (IDH) and two enzymes in the pentose phoshate pathway (PPP), glucose 6-phosphate dehydrogenase (G6PD) and 6-phosophogluconate dehydrogenase (6PGD); [34]; reviewed in [35]. Recent work in *Drosophila* has demonstrated significant network-like interactions between IDH, MEN and G6PD, in which lowered activity of one can induce compensatory changes of the other two [33], [34]. In these studies the variation in the magnitude of response to reduction in enzyme activity was proposed to reflect the different contribution of each enzyme to the standing cellular NADPH pool under benign conditions.

The NADP^+^ reducing enzymes have been found to increase in activity under conditions of oxidative stress, presumably to produce additional NADPH for ROS clearance [26], [30], [31], [36]. Consistent with these findings, over-expression of G6PD extends longevity and enhances resistance to oxidative stress in *Drosophila* [27]. Combined, these results suggest that increased NADPH production would be a viable compensatory strategy to combat ROS in SOD1-null flies. Conversely, several independent microarray analyses report that carbohydrate utilization is generally suppressed under conditions of oxidative stress, while lipid catabolism is enhanced [37], [38], [39] (TLP, unpublished). The finding that CPT1, an enzyme responsible for importing long-chain fatty acids into the mitochondria for β-oxidation, becomes essential in the absence of SOD1 activity [40] also supports a hypothesis of increased reliance on lipid catabolism. NADPH production may therefore be requisite to maintain lipid homeostasis under oxidative stress.

With respect to NADPH function, the current model of the metabolic response to oxidative stress is therefore somewhat paradoxical. A shift in which carbohydrate utilization is generally reduced in favor of lipid catabolism would be expected to impair the generation of NADPH, while substantial amounts of NADPH would simultaneously be required for the maintenance of lipid homeostasis and GSH levels. The logical result of this apparent conflict is the main hypothesis for the current study: if organisms are under any long-term condition of oxidative stress then both NADPH and lipid reserves should be increasingly depleted. SOD1-null organisms provide just such a case of chronic oxidative stress and are thus a valuable model system in which to investigate the significance of NADPH in the interplay between ROS-metabolism and energy utilization.

**Figure 1:**
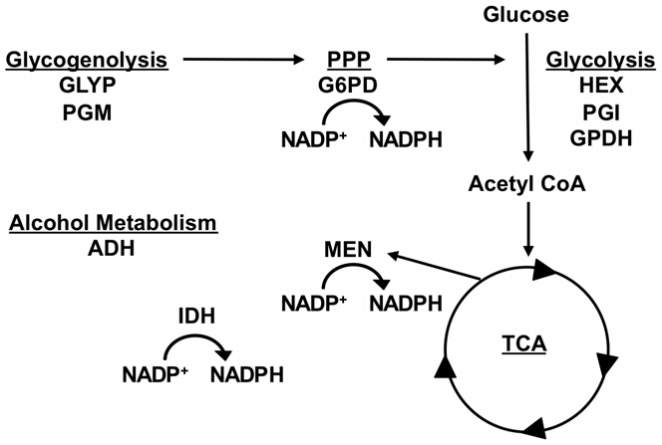
Schematic representation of metabolic pathways and the positions of the enzymes studied. See text for abbreviations.

Here we present a study of the role of NADPH as a central mediator of physiological responses to oxidative stress caused by the SOD1-null allele. In a comparison of a SOD1-null to a transgenic rescue line, the activities of a wide range of enzymes involved in carbohydrate metabolism (Figure 1) were found to be down-regulated throughout the adult lifespan of the SOD1-nulls. The NADP^+^-reducing enzymes were found to have significantly lower activity than the remainder of the enzymes assayed, contrary to our expectations. This down-regulation is consistent throughout the lifespan of SOD1-nulls, suggesting that the lowered enzyme activities are not simply a product of aging or oxidative damage accumulated in the adult fly. The steady-state concentration of NADPH was also lower in SOD1-nulls, but we noted no concurrent change in triglyceride concentration and an increase in total lipid concentration. Based upon the somewhat unexpected results of these analyses, we expanded our investigations to further examine enzyme activities, lipids and triglycerides under starvation conditions, conditions chosen to directly impact triglyceride and lipid stores. We found, surprisingly, that SOD1-null mutants are relatively tolerant to starvation stress – significantly more so than SOD^+^ control flies. We speculate that this initially counterintuitive, albeit relative, tolerance to starvation in the SOD-nulls is likely due to the already dampened carbohydrate metabolism or associated metabolic compensations observed in this genotype. Under starvation conditions SOD1-nulls were able to metabolize lipids, suggesting that the lack of lipid metabolism we observe under benign conditions is not simply a function of oxidative damage preventing the breakdown of lipids. From the results presented here, we propose a model in which nutrient sensing pathways respond to increased superoxide to reduce carbohydrate metabolism and maintain storage of lipids in an effort to limit products entering the ETC, thus limiting the production of ROS.

## Results

### Enzyme Activities, Triglycerides and Lipids Under Benign Conditions

**Figure 2:**
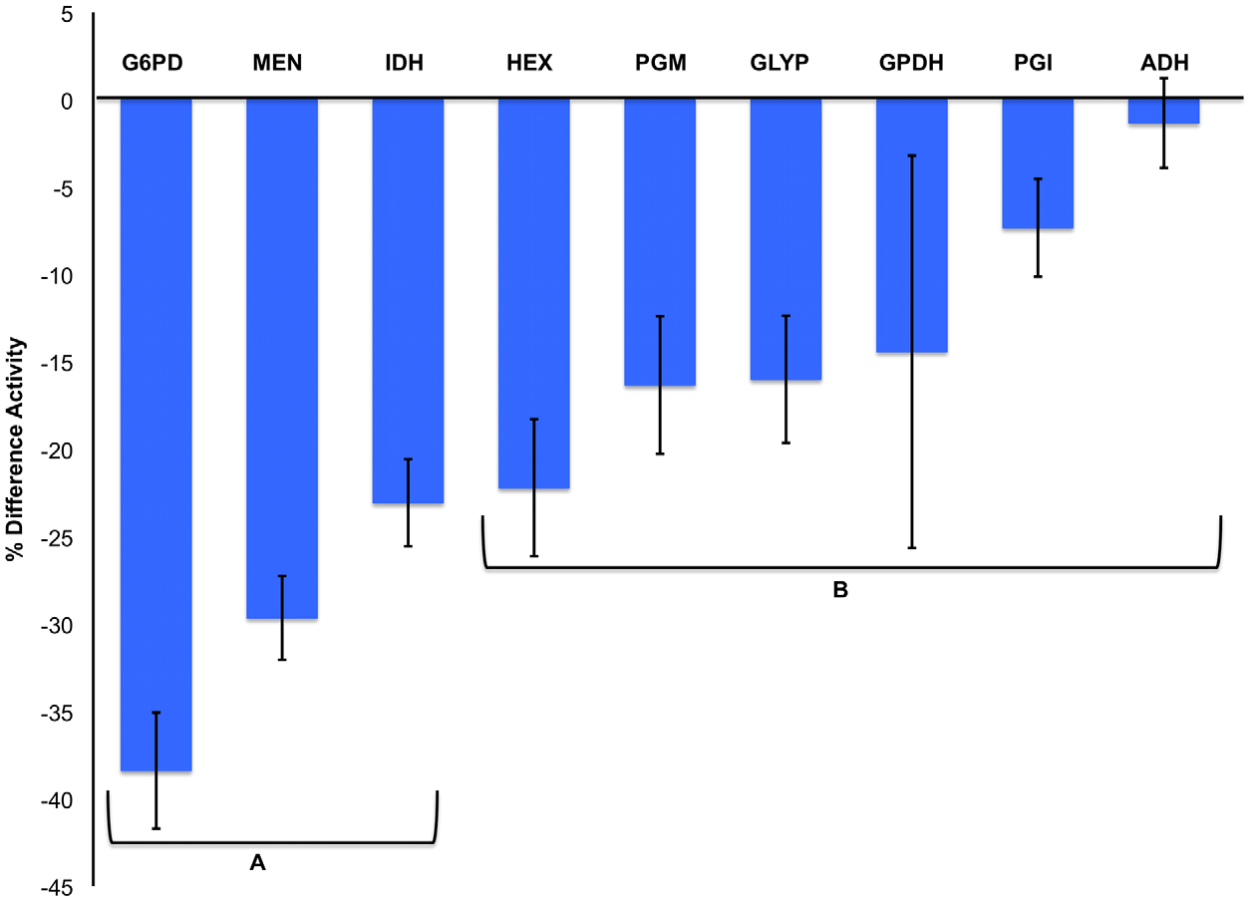
Percent difference of enzyme activities of SOD1-nulls compared to SOD^+^ controls under benign conditions. See Table 1 for mean ± SEM and statistics. Bars not connected by the same letter code are statistically different, as determined by one-way ANOVA Contrast, F1,81 = 26.36, p<0.0001.

A cross section of enzymes involved in carbohydrate metabolism (G6PD, MEN, IDH, alcohol dehydrogenase [ADH], hexokinase [HEX], phosphoglucomutatse [PGM], phosphoglucose isomerase [PGI], glycogen phosphorylase [GLYP], and glucosephoshate dehydrogenase [GPDH]) was assayed in order to determine the impact of the SOD1-null mutation on carbohydrate metabolism. Unexpectedly, we found that under benign laboratory conditions, the SOD1-null allele resulted in an overall reduction of metabolic activity: homozygous SOD1-null flies had significantly lower activity of all metabolic enzymes assayed compared to SOD^+^ controls (Table 1, Figure 2). Enzyme activities of SOD1-null flies were 1.6–39.1% lower than SOD^+^ controls (Table 1, Figure 2). The NADP^+^ reducing enzymes G6PD and MEN showed the largest differences in activity between the two genotypes, with 39.1% and 33.0% differences, respectively (Figure 2). The smallest significant difference in activity was in the enzyme ADH, which is considered to be ancillary to the core of carbohydrate metabolism, suggesting that the highest impact of the SOD1-null allele is on those enzymes most centrally involved.

**Table 1:** Statistical analysis of enzyme activity levels of 4–6 day old SOD1-null and SOD+ control flies under benign conditions.

Enzyme	Mean SOD1-null Activity ± SEM	Mean SOD+ control Activity ± SEM	% Difference	p
G6PD*	3.62±0.133	5.93±0.209	−39.1	<0.0001
MEN*	5.10±0.130	7.29±0.213	−33.0	<0.0001
HEX*	3.97±0.174	5.14±0.139	−22.7	0.0002
IDH*	5.72±0.146	7.47±0.188	−25.7	<0.0001
PGM*	25.7±0.766	31.0±0.649	−17.0	<0.0001
GLYP*	16.4±0.321	19.8±0.795	−17.1	0.0002
GPDH	2.13±0.271	2.47±0.107	−14.1	0.2275
PGI*	62.8±1.25	68.0±0.956	−7.63	0.0006
ADH	14.4±0.267	14.7±0.195	−1.55	0.2119

Mean (OD units) ± SEM is given for each enzyme for both SOD1-null and SOD+ control. ANCOVA was performed for each enzyme to determine statistically significant differences between SOD1-nulls and SOD+ controls. Significant differences are denoted by ‘*’. The ‘% Difference’ describes the difference in enzyme activity of SOD1-nulls compared to SOD+ controls.

**Figure 3:**
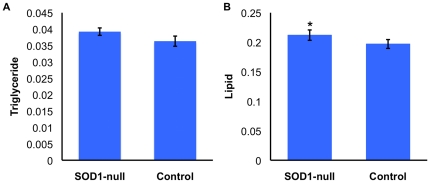
Mean triglyceride and lipid concentrations for SOD1-null and SOD^+^ control flies under benign conditions. (A) Triglyceride concentration is expressed as the mean triglyceride concentration (mmol/L standardized per mg wet weight) ± SEM for each genotype: SOD1-null 0.039±0.0011, Control 0.036±0.0015. ANCOVA F_2,17_ = 1.43, p = 0.1096. (B) Lipid concentration is expressed as mean mg lipid ± SEM for each genotype: SOD1-null 0.212±0.088, Control 0.197±0.0075. ANCOVA F2,22 = 3.36, p = 0.0498. Significant differences denoted as: ‘*’ indicates p<0.05.

Lipid and triglyceride concentrations were quantified to determine if large differences in activity of NADPH producing enzymes (IDH, G6PD and MEN), between SOD1-nulls and SOD^+^ controls, affected the production and storage of lipids as suggested in previous studies [33], [34]. These processes are known to be dependent on NADPH as a reducing cofactor [41], [42], [43]. A number of models in the literature also indicate that, under oxidative stress, lipids may be preferentially metabolized for energy [37], [38], [39]. Somewhat surprisingly, we find a small but statistically significant increase in the concentration of storage lipids in the SOD1-nulls relative to the control flies (Figure 3b). We find no significant difference in the concentration of soluble triglycerides (Figure 3a), although the trend suggests a higher concentration in SOD1-nulls. These results were highly unexpected, as previous evidence points to increased reliance on lipids for energy, yet our results in this experiment offer no evidence in support of such a reliance.

**Figure 4:**
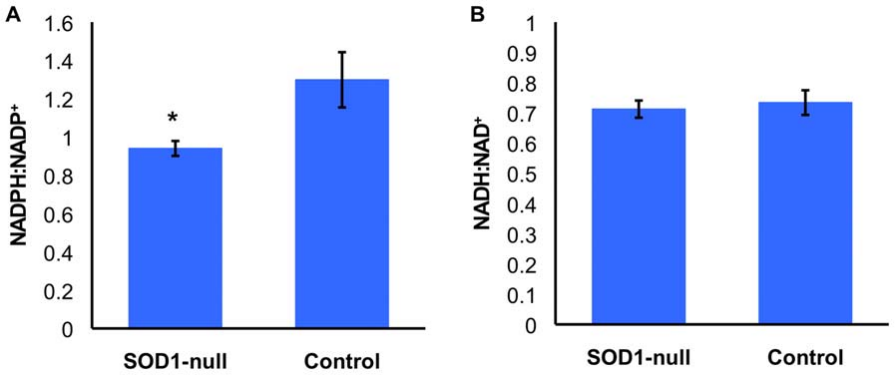
Metabolite concentrations of SOD1-nulls compared to SOD^+^ controls under benign conditions. The data are expressed as mean relative absorbance ± SEM. Significant differences were determined by ANCOVA. (A) NADPH:NADP^+^, F_1,8_ = 4.807, p = 0.0485, (B) NADH:NAD^+^, F_1,8_ = 0.116, p = 0.892. Significant differences denoted as: ‘*’ indicates p<0.05.

Due to large decreases in the activity of the NADP^+^-reducing enzymes, and the role of NADPH in lipogenesis, HPLC analysis was conducted to determine the relative concentrations of nicotinamide cofactors. Consistent with the 22.7–39.1% lower activity of NADP+-reducing enzymes in the SOD1-nulls, the concentration of NADPH is 28.8% lower in SOD1-nulls. This result is consistent with earlier speculation as to the relative contributions of the NADP^+^-reducing enzymes [33], [34], where MEN is proposed to contribute the most NADPH, and G6PD the least. The ratios of the reduced and oxidized forms of metabolites are often used to represent the cellular condition; the NADH:NAD^+^ ratio has been demonstrated to increase as *Drosophila* age [44], with a high ratio indicating a very oxidative cellular environment, and the ratio of NADPH:NADP^+^ representing the reductive cellular environment that is expected to decrease with age [44]. As expected the NADPH:NADP^+^ ratio of SOD1-null flies is significantly lower than that of SOD^+^ controls (Figure 4, panel A), due to a lower concentration of NADPH (Table 2). Contrary to our expectations, SOD1-null flies have a lower concentration of NADH as compared to SOD^+^ controls (Table 2), however this does not translate into a lower ratio of NADH:NAD^+^ (Figure 4, panel B).

**Table 2:** Statistical analysis of cofactor concentrations of 4–6 day old SOD1-null and SOD+ control flies under benign conditions.

Enzyme	Mean SOD1-null Absorbance± SEM	Mean SOD+ control Absorbance± SEM	% Difference	p
NADP+	9299.4±260.4	9752.83±856.63	−4.65	0.5386
NADPH*	8729.7±407.7	12257.4±830.9	−28.8	0.0072
NAD+	14938.8±753.797	16655.6±2420.27	−10.3	0.5170
NADH*	10555.8±242.239	12049.6±357.120	−12.4	0.0225

Mean (relative absorbance) ± SEM is given for each cofactor for both SOD1-null and SOD+ control. ANCOVA was performed for each cofactor to determine statistically significant differences between SOD1-nulls and SOD+ controls. Significant differences are denoted by ‘*’. The ‘% Difference’ describes the difference in absorbance of SOD1-null samples compared to SOD+ control samples.

### Enzyme Activities, Triglycerides and Lipids Under Starvation

**Figure 5:**
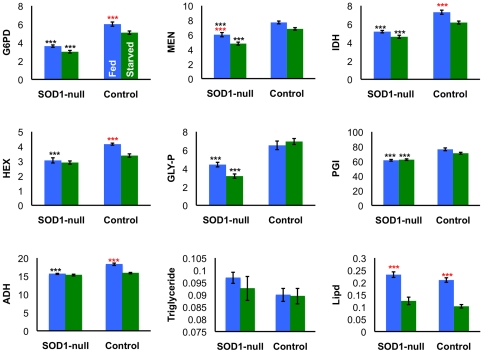
Enzyme activities, triglyceride and lipid concentrations of SOD1-nulls and SOD^+^ controls before (blue bars) and after 24 hours starvation (green bars). Activity data (A–F) are presented as mean activity ± SEM. (A) G6PD, F_3,36_ = 61.630, p<0.0001, (B) MEN, F_3,36_ = 31.615, p<0.0001, (C) HEX, F_3,36_ = 20.634, p<0.0001, (D) IDH, F_3,36_ = 45.405, p<0.0001, (E) PGI, F_3,36_ = 27.415, p<0.0001, (F) ADH, F_3,36_ = 33.825, p<0.0001, (G) GLYP, F_3,36_ = 16.595, p<0.0001, (H) Triglyceride concentration is expressed as the mean triglyceride concentration (mmol/L) ± SEM, F_3,36_ = 1.023, p = 0.3939, (I) Lipid concentration is expressed as mean mg lipid ± SEM, F_3,36_ = 32.237, p<0.0001. Significant differences denoted as: ‘*’ indicates p<0.05, and ‘***’ indicates p<0.0001, as determined by one-way ANOVA and Tukey's HSD; black stars indicate differences between genotypes (SOD1-null versus SOD^+^ control), red stars indicate differences between experimental conditions within a genotype (e.g. fed and starved SOD^+^ control flies).

**Figure 6:**
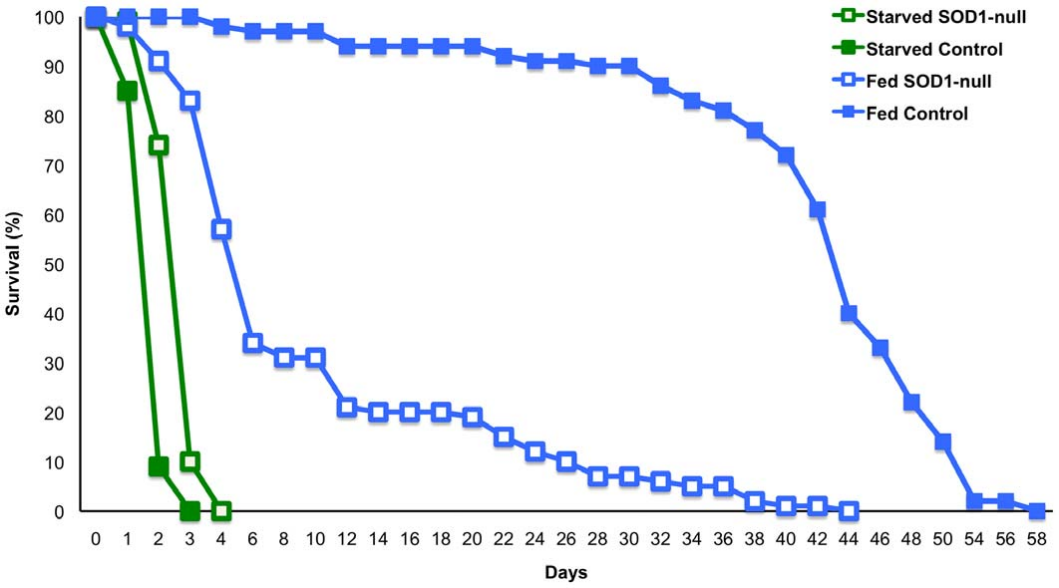
Survivorship of SOD1-nulls and SOD+ controls under starvation. Both genotypes were subjected to starvation and compared to controls fed ad libitum. Their survival was tracked until 100% mortality was reached. Under starvation SOD1-nulls lived significantly longer than SOD+ controls (Kaplan-Meier log-rank, Chi-square: 87.956, p<0.0001 mean SOD1-null lifespan: 2.84 days, SE: 0.062, mean control lifespan: 1.94 days, SE: 0.049).

Given that lower activity of NADP^+^-reducing enzymes is not reflected in the concentration of triglycerides and lipids, we questioned the ability of SOD1-null flies to catabolize these compounds. To confirm that SOD1-nulls are able to upregulate lipid metabolism, typical starvation assays were performed to provide a condition in which metabolism of lipids would be necessary. Furthermore, reductions of metabolic enzyme activity similar to those described above have been proposed as a starvation resistance mechanism [45]; reviewed in [46]. This similarity (Figures 2 and 3) suggested that the SOD1-null flies may be consuming less food than SOD^ +^ flies. If this scenario were the case, lipid stores would be expected to remain relatively constant under a true starvation condition, and at the very least would not be degraded at the same rate as lipid stores in starved SOD^+^ flies. After 24 hours of starvation, both genotypes were again assayed for differences in enzyme activities. Consistent with our expectations, after 24 hours of starvation stress, the SOD^+^ control flies had a significant decrease in activity of all enzymes assayed compared to control flies fed *ad libitum*; reductions in activity ranged from 6.96% to 18.67% (Figure 5, panels A–G). Consistent with activity results under benign conditions, the SOD1-nulls maintain lower enzyme activity under fed and starved conditions for all enzymes, with the exception ADH activity. In contrast to the SOD^+^ controls, SOD1-nulls experienced only small reductions in enzyme activity following starvation (Figure 5, panels A–G), MEN and HEX being the only enzymes significantly reduced. For both genotypes, 24 hours of starvation resulted in significant reductions of overall lipid concentration, however soluble triglycerides remained relatively unaffected (Figure 5, panels H–I). To further investigate differences in starvation tolerance between the two SOD1 genotypes, we quantified and compared survival of both genotypes under starvation stress as compared to control flies fed *ad libitum*. Surprisingly, under starvation the maximum survival of SOD1-null flies is 24 hours longer than SOD^+^ controls (an increase of 33%, Figure 6), in dramatic contrast to the significantly shorter lifespan of the SOD1-null line compared to SOD^+^ controls under benign conditions [13] (Figure 6).

### Age Profile of Enzyme Activities, Triglyceride and Lipid Concentration

**Figure 7:**
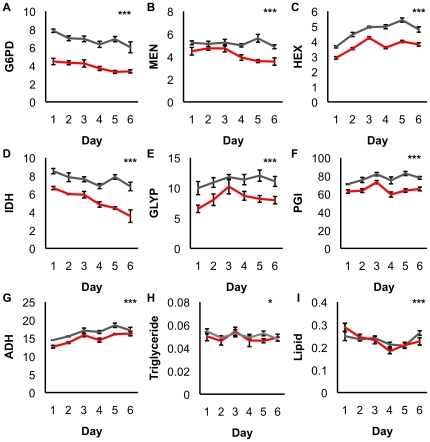
Enzyme activities, lipids and triglycerides were assayed every 24 hours for 6 days in SOD1-nulls (red line) and SOD^+^ controls (grey line). (A) G6PD, F_3,56_ = 99.18, p<0.0001 (B) MEN, F_3,56_ = 15.60, p<0.0001 (C) HEX, F_3,56_ = 32.56, p<0.0001 (D) IDH, F_3,56_ = 65.62, p<0.0001 (E) GLYP, F_3,56_ = 12.77, p<0.0001 (F) PGI, F_3,56_ = 23.0, p<0.0001 (G) ADH, F_3,56_ = 22.80, p<0.0001. Each point in the line represents mean activity (OD units) for 5 samples at that time point ± SEM. (H) Triglyceride concentration, each point in the line represents mean triglyceride concentration (mmol/L standardized per mg wet weight) for 5 samples at that time point ± SEM, F_3,56_ = 0.3857, p = 0.9854 (I) Lipid concentration, each point in the line represents mean lipid concentration for 10 samples at that time point ± SEM, F_3,117_ = 428.90, p<0.0001. Significant differences denoted as: ‘*’ indicates p<0.05, and ‘***’ indicates p<0.0001, as determined by ANCOVA.

In order to confirm that the results observed under benign conditions were consistent throughout the viable lifespan of SOD1-nulls and that differences between genotypes were not inflated based on premature aging in this genotype, age profile experiments were conducted. Enzyme activity, triglyceride and lipid concentration were quantified in sets of male flies every 24 hours for six days. While it is difficult to determine an overall trend across the enzymes, the SOD1-nulls maintained a significantly lower activity of all metabolic enzymes relative to controls throughout the experiment (Figure 7, panels A–G). Similarly, although differences are small (less than 10%) and the values vary from day to day, the SOD1-null triglyceride and lipid concentrations were generally higher than that of controls across the experiment (Figure 7, panels H–I). This result – marginally higher concentrations of lipids in the SOD1-nulls, is consistent with our earlier results (Figure 3, and unpublished): that concentrations of lipids are generally higher, but only to a very small percentage, in SOD1-nulls.

## Discussion

The *D. melanogaster* SOD1-null allele is proposed to present conditions of oxidative stress through the accumulation of superoxide and its derivatives [12], [13], [47]. In the absence of SOD1 activity, the glutathione-dependent antioxidants are thought to be largely responsible for oxidative stress management, utilizing the reducing cofactor NADPH to cycle between reduced and oxidized form [13]. Recent microarray analyses (TLP, unpublished) suggest a shift from carbohydrate metabolism to utilization of lipids for energy in SOD1-null *Drosophila,* and it is well understood that lipid biosynthesis also requires NADPH. Furthermore, preliminary evidence (Janusz-Renton and Parkes, unpublished) suggests that a carbohydrate-rich diet severely impacts survival of SOD1-nulls. These connections suggest an interaction between SOD1 function, carbohydrate metabolizing enzymes, and lipid and NADP^+^/NADPH concentrations. In the work presented here, we document a negative shift in carbohydrate metabolism (Table 1, Figure 2), but find no reduction in lipid concentration in the SOD1-null flies (Figure 3). Importantly, we show that SOD1-null flies do metabolize lipids under starvation stress indicating that the SOD1-null mutation does not eliminate the *potential* for increased lipid catabolism. We suggest that the small, but statistically significant, increase in lipid concentration evident under benign conditions most likely results from an overall reduction in metabolism in SOD1-nulls and hypothesize that this is perhaps a physiological adaptation to reduce ROS production. Consistent with this hypothesis, we find that SOD1-nulls are relatively tolerant to starvation compared to SOD^+^ flies, (Figure 5, panels H–I; Figure 6) perhaps reflecting this same reduction in metabolic rate. An age profile of enzyme activity, triglyceride, and lipid concentration, confirms that the trends observed at 4–6 days of age were consistent over the viable lifespan of the SOD1-nulls and not an artifact of any specific the age of the flies (Figure 7).

### Reducing the production of ROS

Defense against oxidative stress through changes to metabolism and metabolic processes has been documented in a variety of organisms [26], [29], [31], [48]. Here we provide evidence that the activity of a number of carbohydrate metabolizing enzymes is lowered in SOD1-null flies, presumably due to oxidative stress caused by lack of SOD1 activity (Table 1, Figure 2). We hypofthesize that this reduction in overall carbohydrate metabolism is a means to reduce the production of ROS. Additionally, we find that not all enzymes are equally affected by the lack of SOD1 activity under benign conditions. The NADP^+^-reducing enzymes are particularly reduced, with 22.7–39.1% lower activity than controls, and SOD1-null flies have approximately 28.8% less available NADPH (Table 2).

While Merritt *et al*. [34] proposed that G6PD contributed relatively little to the NADPH pool in adult *Drosophila* under benign conditions, other research [18] indicates that modification of G6PD activity can have a major impact on longevity. SOD1-nulls were found to have decreased activity of G6PD by up to 40%, suggesting an important role for this enzyme in the response to the chronic oxidative stress caused by the SOD1-null mutation. We propose that the down regulation of the NADP^+^-reducing enzymes in SOD1-nulls functions to limit metabolic generation of ROS by reducing carbohydrate metabolism and flux through the electron transport chain. It is also possible that the reduced production of NADPH observed in the SOD1-nulls may be a means to decrease the activity of NADPH oxidase (NOX), an enzyme that utilizes NADPH for the production of superoxide. In addition, NADPH functions as a cofactor in the generation of nitric oxide by the enzyme nitric oxide synthase (NOS) [49]. Under conditions of elevated steady-state levels of superoxide, reducing NO generation might be expected to result in enhanced production of highly cytotoxic peroxynitrite. Thus, despite its requirement in maintaining a relatively reduced redox state via its interactions with the thioredoxin system [21], [23], NADPH may paradoxically promote oxidative stress in SOD1-null organisms through the action of these ancillary enzyme systems. It seems more likely, however, that simply down regulating NOX and/or NOS would be a more effective approach to curtailing superoxide/peroxynitrite generation than would a general dampening of NADPH production. The current work cannot distinguish between these two possibilities, however, and future studies will examine the function of NOX and NOS in SOD1-null flies directly.

Strikingly, despite the reduction in enzyme activity, and lower steady state NADPH concentration, we have not detected major downstream effects on lipid or triglyceride concentrations (Figure 3). This result is in apparent contrast to previous studies that found reductions in triglyceride concentration in response to decreases in the activity of NADP^+^-reducing enzymes [32], [33], [34]. Although we found that SOD1-nulls had statistically higher concentrations of lipids across the first 6 days post-eclosion, the small size of this difference makes it difficult to interpret the biological significance of this result. One thing that does appear certain, however, is that a reduction in NADPH concentration, or NADP^+^-reducing enzyme activities, does not necessarily result in a reduction in lipid or triglyceride concentration. It is possible that the reductions observed in other studies resulted from indirect pleiotropic effects, rather than due to a direct role of NADP^+^-reducing enzymes. Investigation of the actual usage of NADPH and NADH in triglyceride and lipid metabolism under various conditions is required to fully understand this relationship and to properly interpret the results of this and other previous studies.

Our results can also be interpreted in light of citrate metabolism and the transporter protein INDY (encoded by *I*'*m Not Dead Yet*) which shuttles excess citrate from the mitochondria to the cytosol [reviewed in [50]. *Drosophila* INDY mutants are long-lived and have an altered metabolism that is similar to flies kept on low calorie medium [51]. Normally, citrate transported to the cytoplasm is converted to isocitrate by cytosolic aconitase [52], also reviewed in [50]. This isocitrate is subsequently metabolized by cytosolic IDH, producing NADPH. Interestingly, the activity of cytosolic aconitase is decreased in SOD1-null mutants [23]. A build up of citrate in the cytosol, due to decreased aconitase activity, has been shown to lead to increased fatty acid synthesis, presumably because citrate is free to react with ATP-citrate lyase [reviewed in [50]. If such an increase in lipogenesis is occurring in the SOD1-nulls, it may be that the metabolic shift towards lipid catabolism we had expected to find evidence of is indeed taking place, but is being compensated for by a commensurate increase in lipid production. This balance would explain both our observation that lipid concentrations are not reduced in SOD1-nulls and the reductions we observe in IDH activity and NADPH concentration in this genotype. Further research is required to determine if this mechanism can explain the reductions seen in the other carbohydrate metabolizing enzymes. A SOD2 mutation has also been shown to decrease aconitase activity in the mitochondria [23] potentially having a similar affect on mitochondrial IDH and NADH production [reviewed in [8]. Future investigations will probe this possible connection between SOD function, citrate metabolism, INDY, and lipogenesis.

### Relative Starvation tolerance of SOD1-nulls

Perhaps the most surprising result presented here is the evidence of relative starvation tolerance in the SOD1-nulls (Figure 6). While this tolerance seems counterintuitive, our results suggest that an overall reduction in metabolism may provide an explanation. Harshman and colleagues [45] reported that starved *Drosophila* had lower activity of 12 enzymes involved in carbohydrate metabolism when compared to unstarved controls [also reviewed in [46]. Many starvation resistant genotypes exhibit a similar strategy and also tend to have greater lipid stores than controls [46]. Here we present evidence for both lowered activity of carbohydrate metabolizing enzymes and potentially increased storage of lipids in SOD1-null *D. melanogaster* (Figure 2 and 3). Although our results cannot unambiguously refute an alternative hypothesis in which SOD1-nulls are consuming less food or selectively consuming specific nutrients, one particular piece of evidence argues strongly against this hypothesis. When SOD1-nulls and controls were put under 24 hours of starvation, we found a significant, and substantial, decrease in lipid concentration in both genotypes. If the SOD1-nulls were feeding less than controls under normal conditions, then we would expect a decreased concentration of storage lipids in the SOD1-nulls throughout the benign condition age profile experiment. No such reduction was observed. An accurate feeding assay, one that does not require pre-starvation of the flies, may give some insight as to the rate of feeding of the two genotypes, or how dietary composition is affecting this phenotype, as seen in Ja *et al*. [53] and Grandison *et al*. [54]. Furthermore, in the current study we did not measure glycogen stores, which if increased in the SOD1-nulls could also result in increased starvation tolerance. However, the activity of GLY-P and PGM were found to have decreased activity in the SOD1-nulls (Figure 2, Table 1) suggesting that these organisms are not drawing heavily upon these stores.

Lack of function mutations in the gene *chico*, which codes for an insulin receptor substrate, result in a similar starvation tolerance to that observed in the SOD1-nulls. Flies with a mutation in *chico* also display increased longevity and elevated levels of SOD1 activity [55], but not SOD2 activity [56]. Although mutations of SOD1 and CHICO have both opposing and similar effects on downstream phenotypes, their pleiotropic effects may indicate that common cell signaling pathways are part of the underlying mechanisms, or at the very least, indicate some degree of communication between the insulin signaling and ROS-metabolism pathways. The recently presented mitochondrial hormesis hypothesis [57] suggests a possible mechanistic explanation for such a connection. This hypothesis was formulated to explain the apparent paradox that although dietary restriction (DR) imparts extended longevity and resistance to a variety of forms of stress, including oxidative stress, empirical evidence has established that DR initially leads to an *increase* in ROS release from mitochondria. The theory purports that this initial burst of excess superoxide into the cytoplasm is detected by a variety of pleiotropic nutrient and stress-responsive signal transduction pathways, including InR and TOR, which subsequently mediate a broad metabolic transition that ultimately reduces energy expenditure and extends longevity under DR conditions. We suggest that high levels of superoxide in the cytosol, derived not from excess mitochondrial production but rather from the lack of cytoplasmic SOD activity, may trigger these same signal transduction pathways and elicit a similar metabolic shift. Thus, SOD1-null mutants may be ‘pre-adapted’ to nutrient deprivation because they constantly experience a metabolic state akin to that normally adopted by organisms only in response to DR.

### Change in carbohydrate metabolism is not associated with age

Age of the fly does not appear to be a contributing factor to the difference in enzyme activities that we observe between knockout and control flies (Figure 7). Our initial experiments were all conducted on 4–6 day old flies, raising concerns that the lower enzyme activities we observed in the SOD1-nulls may be the result of a progressive decline with age – perhaps reflecting an accelerated aging process in the oxidatively stressed flies. To test this, we measured enzyme activity, and triglyceride and lipid concentrations in 1 to 6 day old flies (Figure 7). We observed that SOD1-null enzyme activities are significantly lower than controls throughout their viable lifespan. Furthermore, we find no effect of decreased concentration of NADPH on lipid or triglyceride concentration over the viable lifespan of the SOD1-nulls. These results indicate that the relationships which we observe at 4–6 days of age under benign laboratory conditions are consistent and not a product of cumulative oxidative damage in the adult fly.

### Conclusion

In the current study we provide additional evidence for the pleiotropic impacts of genes involved in ROS-metabolism and for the newly described mitohormesis theory. Our results indicate a model in which organisms under extreme oxidative stress limit the production of ROS through the down regulation of carbohydrate metabolism, resulting in a reduction of products entering the electron transport chain. We provide evidence that available NADPH is still used to produce lipids in the SOD1-null condition despite decreased activity of NADP^+^-reducing enzymes and concomitant decrease in available NADPH. It appears that a fine balance of slowing the production of ROS and maintaining NADPH reducing power is essential under these conditions. Our results provide insight into the relationship between ROS metabolism and nutrient utilization and sensing, and present SOD1-null mutants as an important model system in which to further investigate these connections. More importantly these results indicate the moderation of carbohydrate metabolism in response to increased superoxide, which may be evolutionarily conserved or part of a more general stress response.

## Materials and Methods

### Stocks

Details of the SOD1-null and SOD^+^ control alleles used in this study have been described earlier [12], [13], [47]. In brief, the *D. melanogaster* SOD1-null allele, *cSOD^n10^* ^8^, was originally generated via ethyl methanesulfonate mutagenesis [47]. This allele was introduced into the recipient strain *Oregon R* to create the *SOD1*-null line *w^+^; T0/T0; cSOD^n108^red/TM3*. Our SOD^+^ control genotype is a whole organism transgenic rescue line (*w^+^*; *T5/T5; cSOD^n108^red/TM3*), it was constructed in the same genetic background and has a second chromosome SOD transgene under the control of the native *SOD1* promoter; the line has approximately 50–60% of wild-type SOD1 activity [13]. Despite lower than wild-type activity the *T5/T5; cSOD^n108^red/TM3* control line is phenotypically indistinguishable from wild-type [13], and thus is considered as a control line here. A more detailed description of the lines can be found in [13].

### Fly Handling

To control for larval density dependent effects on enzyme activity, three male and five female *D. melanogaster* were placed in vials containing cornmeal-yeast-agar *Drosophila* medium (CYA; medium contains cornmeal, yeast extract, corn syrup, water and agar, live yeast is added to the surface of food vials as a source of protein) for 48 hours at 25^o^C, in a 12 hour light:dark cycle (12L:12D) and allowed to mate freely and lay eggs. Adult male progeny were collected by lightly anesthetizing with CO_2_ and collecting 0–48 hour old males of a desired genotype (e.g. *w^+^*; *T0/T0; cSOD^n108^red/; cSOD^n108^red*). Collected flies were pooled in new vials and aged for 4 days at 25°C (12L:12D) on CYA. After aging, samples of male flies were handled in a manner specific to the assay: flies for enzyme activity or metabolite assays were frozen at −80°C, flies for starvation assays were transferred to the appropriate media. All fly samples were weighed to the nearest 0.01mg with a precision balance (MX5 Balance, Mettler Toledo AG, Greifensee, Switzerland) prior to analysis and sample weights used as covariates in statistical analyses, unless otherwise stated.

### Starvation Treatment

Groups of twelve males were placed in vials containing either CYA medium (control) or 0.9% agar (starvation) and held at 25°C with a 12L:12D cycle for 24 hours. Post-treatment flies were collected and held at −80°C until processing. Samples were thawed on ice and prepared as detailed below for enzyme activity, triglyceride and lipid concentration. To determine survivability under these conditions, the number of surviving flies was recorded every day until no starved flies survived, after which the number of surviving flies was recorded every other day. Flies were transferred to fresh medium every 4 days.

### Age Profile Sample Collection

Male flies of a desired genotype were collected at 0–24 hours post-eclosion and aged in vials containing CYA medium. Samples of 4 males were collected every 24 hours and frozen at −80°C for later analysis to create an age profile of enzyme activities, triglyceride and lipid concentration.

### Spectrophotometric Enzyme Activity Assays

Samples were prepared and homogenized as previously described [34], [58], [59]. Samples were homogenized in 100 µL of buffer solution per fly (0.1M TRIS-HCl, pH 7.4, 1mM NADP^+^ for G6PD, IDH, MEN and GLYP; 10mM KH2PO4, pH 7.4, 1mM EDTA for ADH, HEX, PGI and PGM), centrifuged at 13,000 rpm at 4°C for twelve minutes and the supernatant collected. Reactions were carried out in a standard 96-well microtiter plate; each reaction contained 10 µL of homogenate supernatant and 100 µL of assay solution per well, with the exception of G6PD and GLYP, which contained 20 µL sample per 100 µL of assay solution. Absorbance was measured with a spectrophotometer (SpectraMax Plus 384, Molecular Devices, Sunnyvale, CA, USA) at 340nm every nine seconds for three minutes (5 minutes for G6PD) at 25°C. Each sample was assayed three times and the mean activity was used for statistical analyses. Assay solutions were prepared according to Table 3.

**Table 3:** Enzyme activity assay solutions were created according to the table.

Enzyme	Reagents
**ADH**	M TRIS-HCl, pH 8.6, 4 mM NAD+, 0.2 M iso-propanol
**G6PD**	20 mM TRIS-HCl, pH 7.4, 0.2 mM NADP+, 3.5 mM G6P
**GLYP**	50 mM KH2PO4, pH 6.8, 0.6 mM NADP+, 7 mg/ml glycogen, 15 mM MgCl2, 5 µl G 1–6 bP, 2 mM 5′AMP, Coupling Enzymes:, 2 U/ml PGM, 2 U/ml G6PD
**HEX**	20 mM TRIS-HCl, pH 8.0, 1 mM NADP+, 100 mM glucose, 2 mM MgCl2, 8 mM ATP, Coupling Enzyme: 0.5 U/ml G6PD
**IDH**	0.1 M TRIS-HCl, pH 8.6, 0.1 mM NADP+, 1.37 mM Isocitrate, 0.84 mM MgSO4
**MEN**	0.1 M TRIS-HCl, pH 7.4, 0.34 mM NADP+, 10.0 mM Malate, 5.0 mM MnCl2
**PGI**	20 mM TRIS-HCl, pH 7.4, 1 mM NADP+, 2.5 mM F6P, 8mM MgCl2, Coupling Enzyme: 0.5 U/ml G6PD
**PGM**	20 mM TRIS-HCl, pH 7.4, 0.5 mM NADP+, 0.83 mM G1P, 1 mM MgCl2, Coupling Enzyme: 0.62 U/ml G6PD

### Triglyceride Content

Soluble triglyceride concentration was determined using the Genzyme Triglyceride –SL Kit (Genzyme Diagnostics P.E.I Inc., Charlottetown, P.E.I, Canada) following the manufacturers suggested dual wavelength protocol monitoring absorbance at A500nm/A660nm (primary/secondary) and subtracting the absorbance at 500nm from the absorbance at 660nm. Values were standardized to mmol/L using the manufacturers suggested standardization procedure. Samples were homogenized as for enzyme assays in the standard 0.1M TRIS buffer described above. Each reaction contained 10 µl of sample and 75 µl assay solution, and was incubated at 37°C for five minutes. Each sample was assayed three times and the mean used for statistical analysis.

### Total Lipid Content

Total lipid content was determined using **t**he protocol for organic lipid extraction from Marron *et al*. [60]. In brief, samples of 4 male flies were weighed (Sample Weight), placed into small glass test tubes and dried overnight at 50°C, after which samples were weighed again (Dry Weight). One milliliter of ether was added to each sample, tubes were corked and left overnight at room temperature to extract lipids. After extraction ether was removed and samples were again dried overnight at 50°C to evaporate any residual ether, and finally weighed again to determine the sample's lipid-free weight (Extracted Weight). The lipid content of each sample was calculated by subtracting the Extracted Weight from the Dry Weight.

### Metabolite Analysis by HPLC

The concentration of nicotinamide metabolites was determined using HPLC analysis and a modified version of the protocol developed by Singh *et al*. [30] for cultures of the bacteria *Pseudomonas fluorescens*. Six male flies were collected into tubes on ice and frozen at −80°C for up to one month. Upon thawing, samples were weighed, homogenized in 1mL of 10mM KH_2_PO_4_ (pH 7.4) buffer, and heated to ∼100°C for 10 minutes to inactivate proteins. Homogenates were centrifuged at 10,000 rpm at 4°C for 10 minutes and filtered into HPLC vials for analysis with a Waters (Milford, MA, USA) Alliance HPLC using a C18 reverse-phase column (Synergi Hydro-RP; 4 µm; 250×4.6mm, Phenomenex, Torrance, CA, USA) and a Waters model 2487 UV-Vis dual-wavelength detector. For detection, a mobile phase of purified and deionized water (Milli-Q, Millipore Corporation, Billerica, Massachusetts, U.S.A.) 20 mM KH_2_PO_4_ and 5% acetonitrile, (pH 7.0) filtered through Millipore filters (Millipore Corporation, Billerica, Massachusetts, U.S.A.). Flow rate of mobile phase was 0.2 ml/min and absorbance was measured at 254nm for detection of NAD^+^, NADH, NADP^+^, NADPH. Detection times for the metabolites were approximately 11.9 minutes for NADP+, 12.5 minutes for NADPH, 20.5 minutes for NAD^+^ and 22.3 minutes for NADH.

### Data Analysis

For assays of benign enzyme activities, lipid and triglyceride concentration, each genotype was replicated 10 times by collecting male flies from 10 to 20 vials, pooling these flies for aging and then collecting 10 sets of 4 flies. Percent differences in enzyme activity (Figure 2) were determined by calculating the difference in activity for each enzyme between SOD1-nulls and SOD^+^ controls across the data sets; statistical analysis was then carried out using those differences. For HPLC analysis of each genotype, 5 replicate samples were assayed. For Age Profile analysis, each genotype was replicated 5 times at each time point. Longevity under starvation analysis was conducted using 100 flies per genotype per treatment. Each measure, with the exception of lipid concentration and longevity, was taken three times for each sample and the mean value used for that sample in statistical analysis. ANCOVAs using sample wet weight as a covariate were conducted for enzyme activities, lipid, triglyceride and cofactor concentrations under benign conditions. Because starvation leads to significant changes in body mass, wet weight was not used as a covariate in the starvation experiments and a one-way ANOVA and Tukey's HSD post-hoc test were used to determine significant differences instead. Analysis of age profile data consisted of ANCOVAs carried out using both sample weight (as in previous assays) as well as day of measurement as covariates. For longevity under starvation Kaplan-Meier (Univariate Survival) log-rank analysis was conducted. All statistical analyses were conducted using JMP 8.0 software (SAS Institute, Cary, NC, USA).
